# Computed Tomography-Based Machine Learning Differentiates Adrenal Pheochromocytoma From Lipid-Poor Adenoma

**DOI:** 10.3389/fendo.2022.833413

**Published:** 2022-03-21

**Authors:** Haipeng Liu, Xiao Guan, Beibei Xu, Feiyue Zeng, Changyong Chen, Hong ling Yin, Xiaoping Yi, Yousong Peng, Bihong T. Chen

**Affiliations:** ^1^ Department of Radiology, Xiangya Hospital, Central South University, Changsha, China; ^2^ National Clinical Research Center for Geriatric Disorders, Xiangya Hospital, Changsha, China; ^3^ Department of Urology, Xiangya Hospital, Central South University, Changsha, China; ^4^ College of Biology, Hunan University, Changsha, China; ^5^ Department of Pathology, Xiangya Hospital, Central South University, Changsha, China; ^6^ Hunan Engineering Research Center of Skin Health and Disease, Xiangya Hospital, Changsha, China; ^7^ Hunan Key Laboratory of Skin Cancer and Psoriasis, Xiangya Hospital, Changsha, China; ^8^ Department of Diagnostic Radiology, City of Hope National Medical Center, Los Angeles, CA, United States

**Keywords:** adrenal incidentaloma, lipid-poor adenoma, subclinical pheochromocytoma, computed tomography, machine learning, logistic regression

## Abstract

**Objectives:**

To assess the accuracy of computed tomography (CT)-based machine learning models for differentiating subclinical pheochromocytoma (sPHEO) from lipid-poor adenoma (LPA) in patients with adrenal incidentalomas.

**Patients and Methods:**

The study included 188 tumors in the 183 patients with LPA and 92 tumors in 86 patients with sPHEO. Pre-enhanced CT imaging features of the tumors were evaluated. Machine learning prediction models and scoring systems for differentiating sPHEO from LPA were built using logistic regression (LR), support vector machine (SVM) and random forest (RF) approaches.

**Results:**

The LR model performed better than other models. The LR model (M1) including three CT features: CT_pre_ value, shape, and necrosis/cystic changes had an area under the receiver operating characteristic curve (AUC) of 0.917 and an accuracy of 0.864. The LR model (M2) including three CT features: CT_pre_ value, shape and homogeneity had an AUC of 0.888 and an accuracy of 0.832. The S2 scoring system (sensitivity: 0.859, specificity: 0.824) had comparable diagnostic value to S1 (sensitivity: 0.815; specificity: 0.910).

**Conclusions:**

Our results indicated the potential of using a non-invasive imaging method such as CT-based machine learning models and scoring systems for predicting histology of adrenal incidentalomas. This approach may assist the diagnosis and personalized care of patients with adrenal tumors.

## Introduction

Adrenal incidentalomas are defined as masses incidentally discovered on abdominal imaging carried out for reasons other than evaluation of the adrenal glands (1–3). Advances in imaging modalities and increased use of imaging studies have increased the incidence of adrenal incidentalomas (2, 3). Histologically, the most common adrenal incidentalomas in non-cancer patients are adrenal adenoma (75%-80%), myelolipoma (6%), and pheochromocytoma (most commonly the subclinical PHEO, sPHEO) (0.3%-5.1%) (4–7). Subclinical PHEOs should be managed differently from adrenal adenomas because they may have secretory function despite being clinically asymptomatic (8–11). In addition, failure to diagnose sPHEO before surgery or biopsy may lead to an adrenergic storm and life-threatening hemodynamic crisis (12, 13). Most adrenal incidentalomas are easily recognized if they display typical radiological features. However, imaging findings of sPHEOs and lipid-poor adenomas (LPA) are usually atypical and often overlap (12–22). It is imperative to distinguish between sPHEO and LPA prior to intervention to avoid complications.

Computed tomography (CT) is one of the most commonly used imaging methods for evaluating adrenal incidentalomas (17–26). Traditional CT assessment usually explores a single imaging feature such as CT value of the tumor, or a combination of several features such as tumor shape and texture, which may still result in a diagnostic dilemma from time to time (21, 22, 26). For instance, a large multicenter cohort study of PHEOs reported that 99.5% (374/376) of PHEOs had an unenhanced attenuation of CT>10 HU (27). Therefore, it appeared unnecessary to obtain biochemical testing such as plasma free or 24-hour urinary fractionated metanephrines for diagnosis of PHEO if the tumor had unenhanced attenuation of CT>10 HU. However, this CT cut off value (≤10 HU) to rule out PHEO might not be adequate to separate sPHEO from LPA. LPA may have HU > 10 HU because it lacks low-density lipids in the tumor and PHEO may have unenhanced attenuation of ≤10 HU in rare cases. Prior studies have also shown that measuring CT values during contrast washout may help to differentiate sPHEO from LPA (21, 22). However, this method requires multi-phase enhanced CT scan and requires a dedicated CT scanning protocol specific for adrenal incidentaloma (26). Advanced imaging analysis such as radiomics with texture assessment of CT images in unenhanced, arterial and venous phases has been shown to classify adrenal incidentalomas, specifically differentiating malignant from benign adrenocortical tumors (28). However, radiomics requires computational expertise and is not used routinely in clinical practice.

Unenhanced/pre-enhanced CT imaging protocol does not use intravenous contrast, which has the advantage of avoiding radiation from additional enhanced scan and contrast agent-associated risks (17, 18). This is especially important for vulnerable patients such as the elderly, children, and patients with renal dysfunction. In addition, detailed assessment of the pre-enhanced CT not only avoids the additional scanning for intravenous contrast-enhanced CT but also may help to avoid more expensive methods such as magnetic resonance imaging with in-phase and out-of-phase sequences to identify lipid signal drop-off (29), and to avoid unnecessary invasive procedures such as biopsies. Furthermore, unenhanced CT imaging has the advantage of being easy to acquire and standardize, short scanning time and not being affected by factors associated with contrast administration such as injection rate and scan delay time for various phases. It is therefore prudent to develop a diagnostic strategy based on existing pre-enhanced CT images and machine learning methods for differentiating different types of adrenal incidentalomas.

In this study, we assessed the accuracy of a CT-based machine learning method and scoring system for distinguishing sPHEO from LPA. We developed two kinds of logistic regression (LR) models with and without features related to enhanced CT, named M1 and M2, respectively. To facilitate the use of the models, we also developed two scoring systems, S1 and S2, based on M1 and M2, respectively. In addition, we tested the performance of the prediction models and scoring systems utilizing pre-enhanced CT images compared with enhanced CT images. We hypothesized that the machine learning models built with the pre-enhanced CT imaging features could classify sPHEO and LPA with satisfying performance.

## Materials and Methods

### Patients

**Figure 1:**
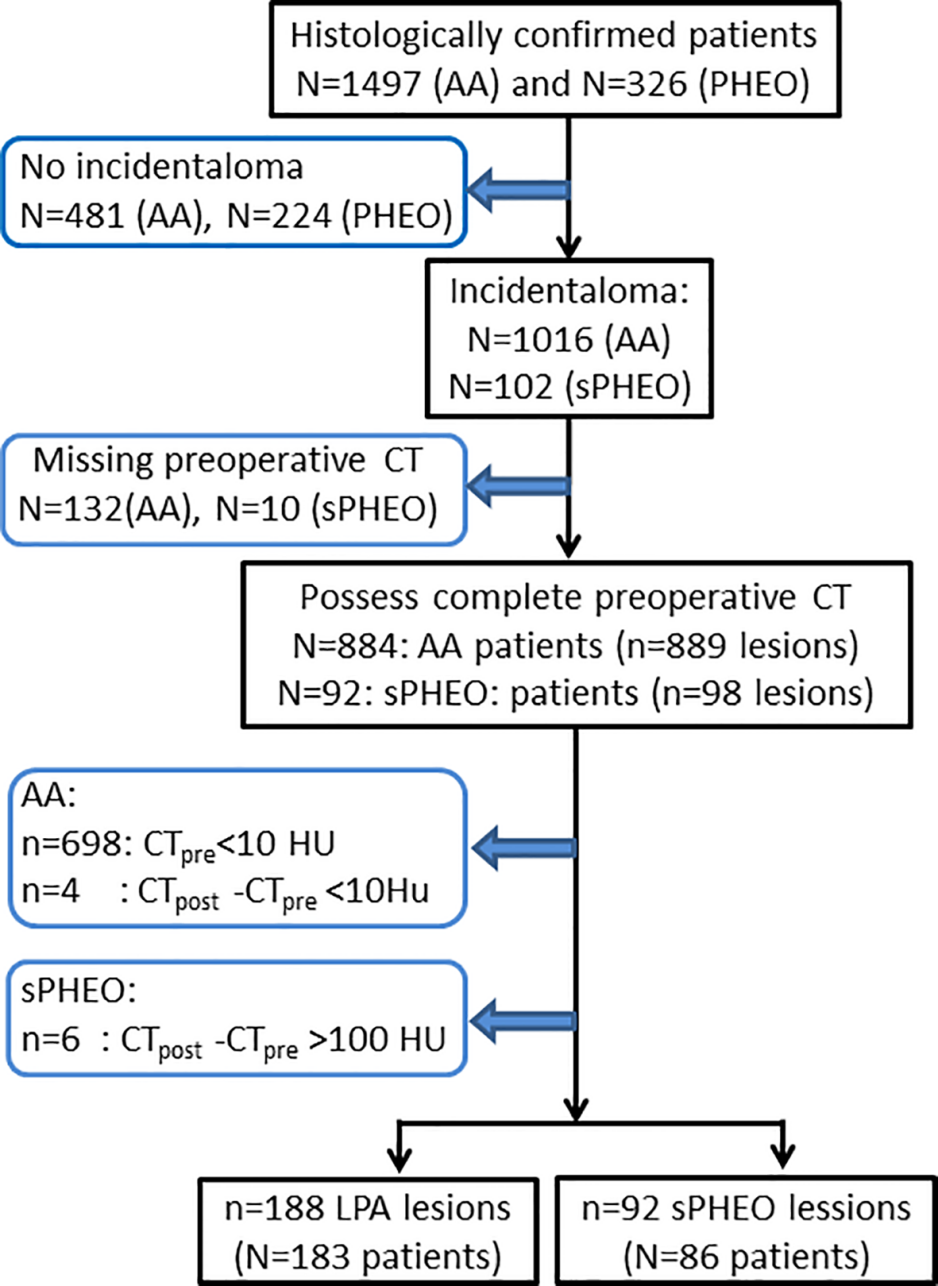
Flow-chart of patient enrollment for this study. CT_pre_, pre-enhanced CT value; CT_post_, enhanced CT value; AA, adrenal adenoma; PHEO, pheochromocytoma; sPHEO, subclinical pheochromocytoma; LPA, lipid poor adenoma; HU, Hounsfield Unit.

Patients with surgical pathology-proven adrenal adenoma or PHEO were identified through searching our institutional medical record database from June 1, 2006 to May 31, 2017. All consecutive patients with detailed medical records as well as pathological results were included in this study. No patients included in this study had adrenal tumor-related therapy prior to the CT scans. The patient recruitment pathway with inclusion and exclusion criteria is presented in **Figure 1**.

The reporting of this study was conformed to the Strengthening the Reporting of Observational Studies in Epidemiology (STROBE) guidelines (30). This retrospective study was approved by Institutional review board of Xiangya Hospital, Central South University, P. R. China and the written informed consents were waived due to the retrospective nature of this study (No.201612638).

### CT Imaging Technique

Patients underwent abdominal CT scans on one of the three CT scanners: a 320-MDCT scanner (Aquilion ONE, Toshiba Medical Systems), a 64-MDCT (SOMATOM Definition, Siemens), or a 16-MDCT (Brilliance 16, Philipps). After routine pre-enhanced CT, contrast-enhanced scans were performed after intravenous administration of 90 to 100 mL of iodinated contrast material (Ultravist 370; Bayer Schering Pharma, Berlin, Germany) at a rate of 3.0 to 3.5 ml/s using a power injector (Ulrich CT plus 150, Ulrich Medical, Ulm, Germany). Enhanced CT images in both arterial and portal-venous phases (scan with fixed delay time of 28 seconds and 65 seconds, respectively) were available for some but not for all patients. The pre-enhanced and contrast-enhanced CT images were reconstructed with a thickness of 1 mm for further evaluation. The scanning parameters are listed in **Supplementary Table S1**.

### CT Imaging Analysis

CT images for each patient were reviewed independently by three abdominal radiologists (XY, CC, FZ) with 10, 20, and 10 years of experience in abdominal imaging, respectively. The three radiologists were blinded to the patient information. CT imaging features of the adrenal tumors included the following: long diameter/dimension (LD, mm), short diameter (SD, mm), pre-enhanced CT value in Hounsfield Unit (HU)(CT_pre_, HU), enhanced CT value (CT_post_, HU), shape (regular or irregular), homogeneity (Homo, homogeneous or heterogeneous) on pre-enhanced CT images, necrosis or cystic degeneration (N/C), calcification (Calc), and contour (sharp or blurred). Consensus in imaging analysis was reached through discussion when differences of opinion existed.

### Statistical Analysis and Predictive Modeling

All statistical analyses were conducted using R (version 3.3.2). For the quantitative features including age, LD, SD, CT_pre_, CT_post_, the Wilcoxon rank-sum test was used to test whether there were significant differences between sPHEO and LPA. Differences in qualitative features including sex, shape, Homo, N/C, Calc, and Contour were analyzed using Chi-square test and Fisher’s exact tests. Three kinds of models, including logistic regression (LR), support vector machine (SVM), or random forest (RF) models were obtained using functions “glm” (in package “stats”), “svm” (in package “e1071”) and “randomForest” (in package “randomForest”), respectively, with default settings. The function “roc.test “(in package “pROC”) was used to compare the area under the receiver operating characteristics (ROCs) curves (AUCs) of the generated models. The score with the largest Youden index, equal to sensitivity + specificity − 1, was defined as the superior cut-off point. Five-fold cross-validations were used to evaluate the performance of these models. ROCs and nomograms were drawn using the functions “plot.roc” (in package “pROC”) and “nomogram” (in package “rms”), respectively. A *P* value < 0.05 was considered statistically significant.

## Results

### Clinical and Radiological Characteristics

**Figure 2:**
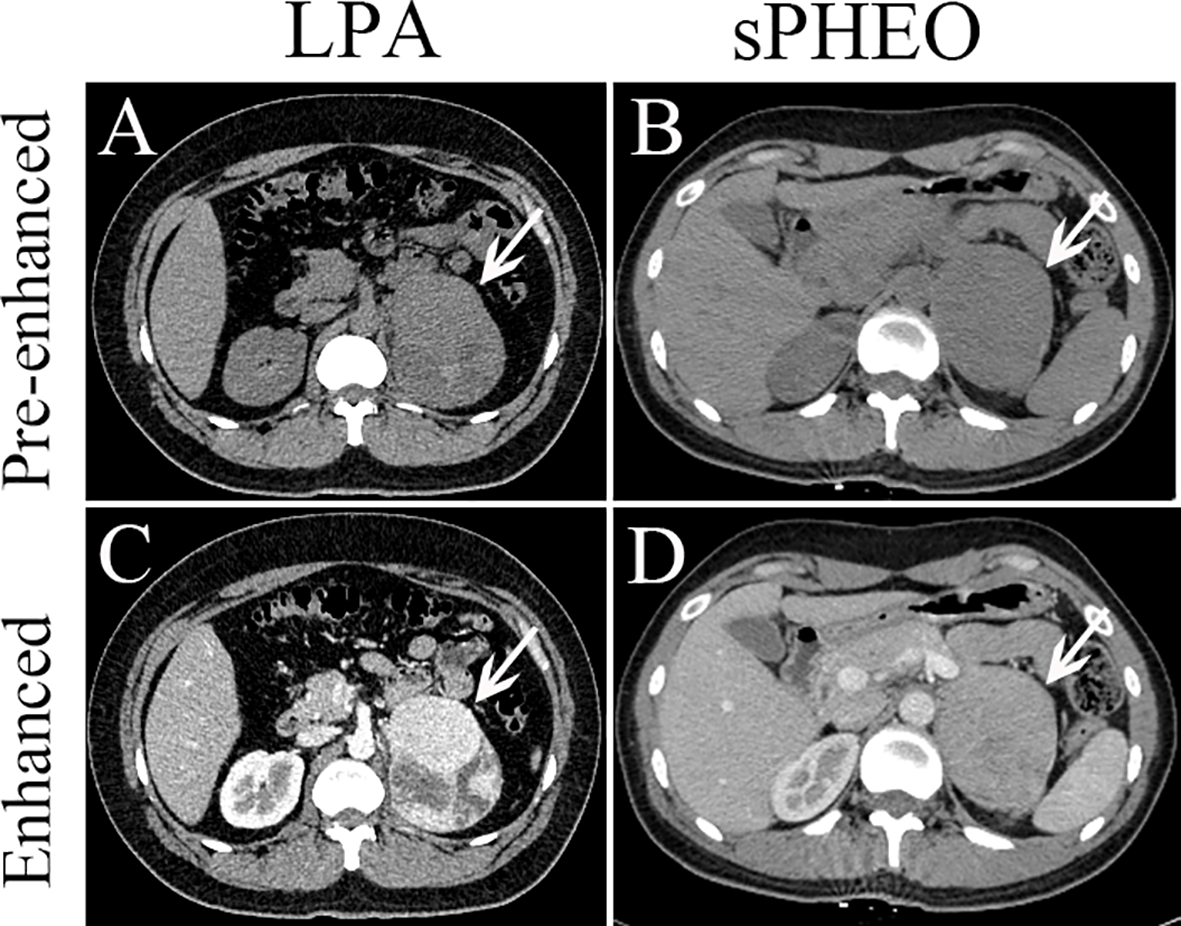
Axial pre-enhanced and enhanced CT images of a patient with lipid-poor adenomas (LPA) (Case 95) and a patient with subclinical pheochromocytoma (sPHEO) (Case 21) showing left adrenal mass at the tumor largest dimensions. On pre-enhanced images, the LPA appeared as an irregular mass with intermediate heterogeneous density **(A)**, while the sPHEO was an elliptical mass with relatively homogeneous density **(B)**. After injection of contrast medium (65s), the LPA was markedly more heterogeneous, with obvious cystic and necrotic areas **(C)**, while the sPHEO showed a mildly heterogeneous enhancement pattern **(D)**.

Comparison between patients with sPHEO and patients with LPA is presented in **Table 1**. A total of 269 patients were retrospectively included in this study. There were 92 tumors in 86 patients with sPHEO and 188 tumors in 183 patients with LPA. No significant differences were found in age, sex, and reasons for CT imaging between the two groups (all *P* > 0.05). Radiologically, many imaging features of sPHEO were overlapped with those of LPA lesions (**Figure 2**). The mean CT attenuation values for both pre-enhanced and enhanced CT of sPHEOs were significantly higher than those of LPAs (*P* < 0.01). In addition, sPHEOs were significantly larger than LPAs in both their long and short dimensions/diameters (*P* < 0.01).

**Table 1:** Clinical and radiological characteristics of subclinical pheochromocytoma (sPHEO) and lipid-poor adenomas (LPA).

Clinical characteristics	LPA	sPHEO	*P*-value
Gender (patients)			0.339^a^
Male	48.1% (88/183)	41.9% (36/86)	
Female	51.9% (95/183)	58.1% (50/86)	
Age (year)	46.6 ± 11.9	47.0 ± 13.2	0.573^c^
Reason for imaging			0.792^b^
Health check	31.7% (58/183)	36.0% (31/86)	
Non-neoplastic diseases	63.9% (117/183)	60.5% (52/86)	
Neoplastic diseases	4.4% (8/183)	3.5% (3/86)	
Location (tumor)			0.053^b^
Left	56.7% (106/187)	42.9% (39/91)	
Right	40.6% (76/187)	50.5% (46/91)	
Bilateral	2.7% (5/187)	6.6% (6/91)	
Imaging findings			
CT value on pre-enhanced images (CTpre) (HU)	23.4 ± 9.7	35.7 ± 8.4	3.90E-19^c^
CT value on post-enhanced images (CTpost) (HU)	64.1 ± 19.7	77.9 ± 26.6	7.74E-06 ^c^
Long diameter (LD) (mm)	28.1 ± 19.1	52.5 ± 22.6	6.02E-19 ^c^
Short diameter (SD) (mm)	22.5 ± 16.1	43.2 ± 17.9	7.57E-20 ^c^
Homogeneity (Homo)			1.97E-18 ^a^
Homogeneous	72.9% (137/188)	17.4% (16/92)	
Heterogeneous	27.1% (51/188)	82.6 (76/92)	
Shape			9.47E-05 ^a^
Regular	79.8%( 150/188)	57.6%(53/92)	
Irregular	20.2% (38/188)	42.4%(39/92)	
Contour			0.105 ^b^
Sharp	99.5% (187/188)	96.7%(89/92)	
Blurred	0.5% (1/188)	3.3%(3/92)	
Calcification (Calc)			0.482 ^b^
No calcification	97.3% (183/188)	95.7% (88/92)	
Calcification	2.7% (5/188)	4.3% (4/92)	
Necrosis or Cystic degeneration (N/C)			
Yes	8.0% (15/188)	71.7% (66/92)	2.15E-28 ^a^
No	92.0% (173/188)	28.3% (26/92)	

^a^Chi-squared test; ^b^Fisher exact test; ^c^Wilcoxon rank-sum test.

HU, Hounsfield Unit.

There was no significant difference in distribution between the two tumor types. On pre-enhanced CT images, the 92 sPHEO tumors showed mildly to moderately heterogenous hyperattenuation, with mildly or intermediately heterogenous (n=66) or relatively homogenous (n=26) enhancement. On pre-enhanced images of the 188 LPA lesions, mild to moderate heterogeneity was found in 51 tumors and a homogenous pattern in 137 tumors. Following contrast-enhancement, mildly/markedly heterogenous (n=149) or homogenous (n=39) enhancement patterns were observed. On enhanced images, N/C imaging feature could be identified in 71.7% (66/92) of sPHEO masses and in 8.0% (15/188) of LPA lesions. Among the sPHEO lesions, 89 had a well-defined margin and the remaining three were ill-defined. Of the 188 LPAs lesions, only one had an ill-defined margin and the remaining 187 were all well-defined.

### Machine-Learning Models

We developed machine-learning models based on seven imaging features that showed significant differences between the two groups of patients: including SD, LD, CT_pre_, CT_post_, Shape, Homo, and N/C. Since a strong correlation was observed between LD and SD (Pearson Correlation Coefficient 0.97, see **Supplementary Files Figure S1**, **S2**), only SD parameter was used to reduce redundancy. Three models were built with three machine learning methods, i.e., LR, SVM and RF. The LR model performed the best among the three models in the five-fold cross-validations, with a prediction accuracy of 0.864 for the validation data (**Supplementary Table S2**). The LR model was considered to be more interpretable than the other two models. Therefore, we used the LR model in the subsequent analysis.

**Figure 3:**
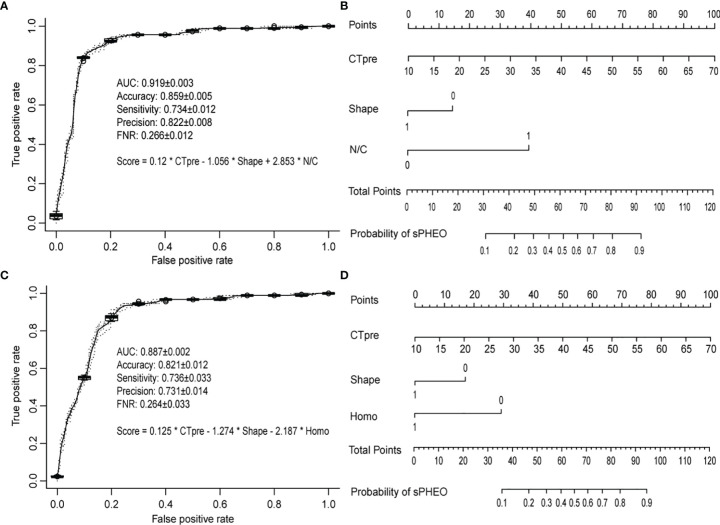
The receiver operating characteristic (ROC) curves and nomograms for the models based on the CT imaging features. The ROC curves were based on predictions from the validation data in five times of cross-validations, while the nomograms were drawn based on predictions from all data used for deriving the model. The average and standard deviation of the predictive performance measures in five times of cross-validations were shown. **(A, B)** refer to model M1 with features of “CT_pre_ + Shape + Necrosis or Cystic (N/C)”; **(C, D)** refer to model M2 with features of “CT_pre_ + Shape + Homogeneity (Homo)”.

To develop a model that could best discriminate sPHEO from LPA, all potential combinations of six features mentioned above were used. A total of 63 models were created, evaluated, and ranked based on the AUCs in five-fold cross-validations (**Supplementary Table S3**). Three parameters were used to select the best model for clinical application: overall performance (high AUC), conciseness (minimum number of features used), and high sensitivity (maximum reduction of missing sPHEO rates). Among the 63 models, the model “CT_pre_ + Shape + N/C” had the largest AUC with only three features used and was therefore, selected as the best model (M1). **Figure 3A** shows the ROC for M1 on the validation data in cross-validations. It achieved an AUC of 0.919 and an accuracy of 0.859, and sensitivity, prediction precision, and a false negative rate (rate of missed diagnosis) of 0.734, 0.822, and 0.266, respectively. M1 performed better than models based on any single feature (**Supplementary Table S4**). The related nomogram for M1 is presented in **Figure 3B**. To determine the probability of sPHEO based on the nomogram, the points for each feature in M1 were obtained by mapping the feature value to the “Points” in the top of **Figure 3B**. “Total Points” were then obtained by summing up the points of features, and mapped to the “Probability of sPHEO” in the bottom of **Figure 3B**.

To facilitate the use of the model, the regression coefficients obtained for each feature in M1 using all data were used to construct a scoring system, which represented the log odds of sPHEO, denoted as the S1 score as follows:


(1) $$\text{S}1\text{ score}=0.12*{\text{CT}}_{\text{pre}}-1.056*\text{Shape}+2.853*\text{N/C}$$

ROC analyses showed that regression coefficients could be simplified without affecting the discriminative accuracy of the score as follows (**Supplementary Figure S3**):


(2) $$\text{S}1\text{ score}=0.1*{\text{CT}}_{\text{pre}}-1*\text{Shape}+3*\text{N/C}$$

Therefore, the total score for S1 was obtained simply by adding 10% of CT_pre_, minus 1 if the shape of the lesion was regular or plus 3 if necrosis or cystic degeneration (N/C) was observed.

The S1 score for all patients ranged from 0 to 7.74 points. An S1 score of 3 was calculated as the optimal cutoff value (**Supplementary Figure S4**), with a sensitivity of 0.892 and a specificity of 0.866 (**Table 2**). This suggested that 89.2% of sPHEO patients would have an S1 score ≥ 3, and the remaining 10.8% would be missed at this cutoff. To reduce the rate of missed diagnosis, lower cutoffs could be adopted. When the cutoff was set at 1, the rate of missed diagnosis could be as low as 1% at the cost of a low precision (43.7%).

**Table 2:** Cut-off values and corresponding performance data for the S1 scoring system based on three CT features, i.e., CT_pre_ + Shape + necrosis or cystic degeneration (N/C), including an enhanced CT feature such as the N/C.

Cutoff	Accuracy	Sensitivity	Precision	Specificity
1	0.578 (0.576, 0.58)	0.989 (0.988, 0.989)	0.437 (0.435, 0.439)	0.377 (0.375, 0.379)
1.5	0.66 (0.658, 0.662)	0.967 (0.966, 0.968)	0.491 (0.489, 0.493)	0.51 (0.508, 0.512)
2	0.768 (0.766, 0.769)	0.956 (0.955, 0.958)	0.591 (0.588, 0.593)	0.676 (0.673, 0.678)
2.5	0.84 (0.839, 0.841)	0.947 (0.945, 0.948)	0.685 (0.682, 0.687)	0.788 (0.786, 0.79)
3	0.875 (0.874, 0.876)	0.892 (0.89, 0.894)	0.765 (0.762, 0.767)	0.866 (0.865, 0.868)
3.5	0.878 (0.877, 0.88)	0.814 (0.811, 0.816)	0.816 (0.813, 0.818)	0.91 (0.909, 0.911)
4	0.865 (0.864, 0.866)	0.74 (0.737, 0.743)	0.831 (0.829, 0.834)	0.926 (0.925, 0.928)
4.5	0.84 (0.839, 0.842)	0.664 (0.661, 0.667)	0.814 (0.811, 0.816)	0.926 (0.925, 0.927)
5	0.83 (0.828, 0.831)	0.611 (0.608, 0.614)	0.826 (0.823, 0.828)	0.937 (0.936, 0.938)
5.5	0.789 (0.788, 0.791)	0.486 (0.483, 0.489)	0.789 (0.786, 0.793)	0.937 (0.936, 0.938)
6	0.765 (0.763, 0.767)	0.349 (0.346, 0.352)	0.842 (0.838, 0.845)	0.968 (0.967, 0.969)
6.5	0.725 (0.723, 0.726)	0.174 (0.172, 0.177)	0.94 (0.936, 0.943)	0.995 (0.994, 0.995)
7	0.699 (0.698, 0.701)	0.087 (0.085, 0.089)	0.999 (0.997, 1)	1 (1, 1)
7.5	0.685 (0.683, 0.687)	0.043 (0.042, 0.044)	0.978 (0.969, 0.987)	1 (1, 1)

### Differentiating sPHEO From LPA Without Enhanced CT Features

Given that enhanced CT may pose increased risks to patient health, such as added radiation dose and potential contrast allergy, we investigated the possibility of distinguishing sPHEO from LPA without the need for features derived from enhanced CT images, i.e. N/C and CT_post_ value. Logistic regression models were developed with four features, and the best model was chosen as described above (**Supplementary Table S5**). The best-ranked model, including the features “CT_pre_ + Shape + Homo”, was named M2. Interestingly, the features included in M2 were similar to those in M1, considering that the features “Homo” and “N/C” both were indicators of tumor texture and structure. **Figure 3C** shows the ROC for M2 on the validation data in cross-validations. M2 achieved an AUC of 0.887, an accuracy of 0.821, sensitivity, precision, and a false negative rate of 0.736, 0.731, and 0.264, respectively. Although the overall performance of M2 was inferior to that of M1 based on the AUC test (**Supplementary Table S6**), the rates of missed diagnosis (i.e., false negative rate) for both models were similar (**Figures 3A, C**). The related nomogram for M2 is shown in **Figure 3D**.

To facilitate the use of M2, the regression coefficients obtained for each feature in the model, based on all data, were used to construct a scoring system without features related to enhanced CT, denoted as S2 Score as follows:


(3) $$\text{S}2\text{ Score}=0.125*{\text{CT}}_{\text{pre}}-1.274*\text{Shape}-2.187*\text{Homo}$$

ROC analyses again showed that regression coefficients could be simplified without affecting the discriminative accuracy of the S2 score, as follows (**Supplementary Figure S5**):


(4) $$\text{S}2\text{ Score}=0.1*{\text{CT}}_{\text{pre}}-1*\text{Shape}-2*\text{Homo}$$

Therefore, total score for S2 was obtained simply by adding 10% of pre-enhanced CT values, minus 1 if the shape was regular or minus 2 if homogeneity was observed.

The S2 score ranged from -2 to 4.74 points. A score of 1 was calculated as the optimal cutoff value (**Supplementary Figure S6**) with a sensitivity of 0.935 and a specificity of 0.773 (**Table 3**). This suggested that 93.5% of sPHEO patients would have an S2 score≥1, and the remaining 6.5% would be missed at this cutoff. Lower cutoffs could be used to reduce the rate of missed diagnosis; when the cutoff was set to -1, the rate of missed diagnosis could be as low as 1% at the cost of reduced precision (41.2%).

**Table 3:** Cut-off values and corresponding performance data for the S2 scoring system based on three CT features, i.e., CT_pre_ + Shape + Homogeneity (Homo), without enhanced CT features.

Cutoff	Accuracy	Sensitivity	Precision	Specificity
-1	0.533 (0.531, 0.534)	0.989 (0.988, 0.99)	0.412 (0.41, 0.414)	0.309 (0.307, 0.311)
-0.5	0.596 (0.595, 0.598)	0.968 (0.966, 0.969)	0.448 (0.446, 0.45)	0.414 (0.411, 0.416)
0	0.692 (0.69, 0.693)	0.968 (0.967, 0.97)	0.517 (0.515, 0.519)	0.556 (0.554, 0.558)
0.5	0.769 (0.768, 0.771)	0.957 (0.956, 0.958)	0.593 (0.59, 0.595)	0.677 (0.675, 0.68)
1	0.826 (0.825, 0.828)	0.935 (0.934, 0.937)	0.669 (0.666, 0.671)	0.773 (0.771, 0.774)
1.5	0.836 (0.834, 0.837)	0.859 (0.857, 0.861)	0.706 (0.704, 0.709)	0.824 (0.822, 0.826)
2	0.824 (0.823, 0.825)	0.738 (0.735, 0.741)	0.727 (0.724, 0.73)	0.866 (0.864, 0.867)
2.5	0.787 (0.785, 0.788)	0.584 (0.581, 0.588)	0.715 (0.712, 0.719)	0.886 (0.885, 0.887)
3	0.769 (0.767, 0.77)	0.404 (0.401, 0.407)	0.788 (0.784, 0.792)	0.947 (0.946, 0.948)
3.5	0.724 (0.722, 0.726)	0.184 (0.182, 0.187)	0.894 (0.89, 0.899)	0.989 (0.989, 0.99)
4	0.699 (0.698, 0.701)	0.086 (0.085, 0.088)	0.999 (0.997, 1)	1 (1, 1)
4.5	0.686 (0.684, 0.687)	0.043 (0.042, 0.044)	0.968 (0.957, 0.979)	1 (1, 1)
5	0.67 (0.668, 0.672)	0 (0, 0)	0 (0, 0)	1 (1, 1)

## Discussion

Adrenal incidentalomas are more commonly seen nowadays due to greater use of clinical imaging and better performance of imaging modalities. Accurate diagnosis is important for proper management of these incidentalomas (31–36). Our previous work on adrenal incidentalomas showed the potential of radiomics and textural features for distinguishing sPHEO from LPA (17, 18). However, radiomics has not been widely used in clinical practice because of its demand on time-consuming computational analysis for high-dimensional features not recognized by human eye. Therefore, the work-up of adrenal incidentalomas still depends mainly on the traditional radiological features assessed *via* visual inspection (14–16, 20–22, 37, 38). In this study, we focused on the traditional radiological features, and our models built with these common CT features had robust performance in classifying these two subtypes of adrenal incidentalomas. Furthermore, our model built with the pre-enhanced CT images performed reasonably well, which is promising for its clinical implication because of its advantages of avoiding radiation exposure and the risks associated with contrast administration.

Our study was unique. We combined the traditional radiological features and machine learning methods to improve the diagnostic performance of non-adrenal CT imaging. Published literature has shown the value of adrenal washout CT and pre-contrast CT in diagnosing PHEO and adenoma. However, there are remaining issues with these CT scan protocols, which still need further research. Adrenal washout CT has a low specificity with a non-negligible proportion of pheochromocytomas mistaken for adenoma (26). The pre-contrast CT of 10 HU as a cutoff is not adequate to separate PHEO and LPA because both can have CT >10 HU (39). Radiomics may play a role in classifying the subtypes of adrenal incidentalomas. Our own radiomic studies on adrenal incidentalomas showed a radiomic signature constructed with CT characteristics and radiomic features, and texture analysis of unenhanced CT images could help to differentiate sPHEO from LPA (17, 18). Additional diagnostic imaging such as 123 I-MIBG and 68Ga-DOTATATE, and biochemical tests checking for elevation of catecholamines and metanephrines could also help to confirm the diagnosis of PHEO but also increase cost.

Interestingly, the best models identified by both scoring systems contained the features CT_pre_ value and Shape, with their respective third feature being similar in nature reflecting internal tumor architecture, i.e., Homogeneity (Homo) for the S1 scoring system and necrosis/cystic degeneration (N/C) for the S2 scoring system. This should not be surprising as these imaging features are commonly scrutinized for differential diagnosis of adrenal incidentalomas (1–7, 19–22, 26). They reflect the textural and structural characteristics, and the biological behavior of the tumors to some extent (2). Although these features are not specific for any particular adrenal tumors and have low diagnostic specificity when used alone, we found that the diagnostic accuracy was substantially increased when these features were used in combination.

Among the imaging features assessed in this study, the feature “N/C” indicating necrosis/cystic degeneration of tumor contributed most to the diagnostic specificity in our prediction model. The “N/C” imaging feature is usually found in PHEOs other than in adenomas despite of more abundant blood supply in PHEOs (10–12, 19–23). It is therefore an imaging feature that prompts a PHEO diagnosis (21, 22). Of note, the “N/C” feature is usually identified on enhanced images. However, our S2 scoring system did not rely on enhanced CT but had similar diagnostic efficiency to the S1 scoring system which included the enhanced CT features. Our results implied that pre-enhanced CT could potentially be used as the imaging choice for differentiating the subtypes of adrenal incidentalomas.

There were several limitations to our study. First, this was a retrospective study from single-center data without external validation, which may reduce the generalizability of our prediction models. A future large-scale prospective multicenter study is needed to validate our results. To the best of our knowledge, this study was the largest case study of these two tumors so far. Nevertheless, the sample size was still modest for a machine-learning study and case selection bias was inevitable due to the retrospective nature of the study. Second, the sensitivity and accuracy for both LR models (M1 and M2) were not high enough for clinical applications. More effective imaging features are needed to improve the model performance. Clinical features including biochemical and pathological tests may also help to distinguish sPHEO from LPA. Third, while the models of M1 and M2 were evaluated with cross-validations, their reliability and performance need to be tested through a well-designed prospective study in a larger cohort with external validation and sufficient statistical power. Additionally, our study was limited for lack of arterial phase and multi-phase scans such as 10-15-minute delayed phase for measuring contrast washout useful for diagnosing adrenocortical adenomas. Lastly, our study was limited for lack of biochemical tests on catecholamines and metanephrines for endocrine secretion data, which could be helpful for differentiating sPHEO from LPA. In addition, we did not have data on autonomous cortisol secretion in our cohort. This was mostly due to incomplete medical records in this retrospective study. A recent longitudinal study of CT attenuation changes indicated the necessity of obtaining autonomous cortisol secretion values to differentiate LPA (>10 HU) from other adrenal incidentalomas as LPA had a reduced cortisol suppression after dexamethasone test, and decreased attenuation values suggesting increased lipid content during follow-up (40). Therefore, we plan to include endocrine data for our future studies of adrenal incidentalomas.

In conclusion, we assessed traditional radiological features on CT images and developed prediction models and scoring systems for distinguishing sPHEO from LPA. Our results suggested that a non-invasive imaging method such as pre-enhanced CT images could be used to predict the histology of adrenal tumors. This approach should assist in the diagnosis and personalized care of patients with adrenal incidentalomas.

## Data Availability Statement

The original contributions presented in the study are included in the article/**Supplementary Material**. Further inquiries can be directed to the corresponding authors.

## Ethics Statement

This retrospective study was approved by Institutional review board of Xiangya Hospital, Central South University and the requirement for written informed consent was waived due to the retrospective nature of this study (No.201612638).

## Author Contributions

XY and YP designed the study. HL, XG, and BX collected and analyzed the clinical data. FZ, CC, and XY performed the imaging analysis. HY reviewed the pathological sides. BC helped in data collection and manuscript editing. HL, XG, and BX wrote the first draft of the manuscript. XY and YP supervised the study and reviewed the manuscript. All authors contributed to the study and approved the final version.

## Funding

This study received funding in part from the Natural Science Foundation of Hunan Province, P.R. China (2018JJ2641), the Xiangya-Peking University, Wei Ming Clinical and Rehabilitation Research Fund (No. xywm2015I35), China Post-Doctoral Science Foundation (2018M632997) and Natural Science Foundation of China (81902727).

## Conflict of Interest

The authors declare that the research was conducted in the absence of any commercial or financial relationships that could be construed as a potential conflict of interest.

## Publisher’s Note

All claims expressed in this article are solely those of the authors and do not necessarily represent those of their affiliated organizations, or those of the publisher, the editors and the reviewers. Any product that may be evaluated in this article, or claim that may be made by its manufacturer, is not guaranteed or endorsed by the publisher.
