# Supplementary material for: Computed Tomography-Based Machine Learning Differentiates Adrenal Pheochromocytoma From Lipid-Poor Adenoma
Source: Front Endocrinol (Lausanne). 2022 Mar 21;13:833413. doi: 10.3389/fendo.2022.833413 (PMC8977471; doi:10.3389/fendo.2022.833413)

**Supplementary files**

**Table S1** Scanning parameters for CT scan

|  | Toshiba  320-MDCT | Siemens  64-MDCT | Philipps  16-MDCT |
| --- | --- | --- | --- |
| Tube current | Automated tube current | 190mAs-centered automated tube current modulation | 250mAs |
| Tube voltage | 120kV | 120kV | 120kV |
| Reconstruction algorithm | Soft tissue standard algorithm | Soft tissue standard algorithm | Soft tissue standard algorithm |
| Slice thickness | 5mm | 5mm | 5mm |
| Slice intervals | 5mm | 5mm | 5mm |
| Reconstruction Matrix | 512×512 | 512×512 | 512×512 |
| Enhanced scan | Dual-phase: 65s, 180s;  Triphasic-phase:28s, 65s, 180-300s | Dual-phase:65s, 180-300s;  Triphasic-phase:28-30s, 65s, 180-300s | Single-phase:65s  Dual-phase:65s, 180-300s |

**Table S2** Performances of machine learning models of logistic regression, support vector machine (SVM) and random forest in five-fold cross-validations on all data used in this study, based on six imaging features that differ significantly between sPHEO and LPA

| Model | Accuracy | Sensitivity | Specificity |
| --- | --- | --- | --- |
| Logistic regression | 0.864 | 0.751 | 0.92 |
| SVM | 0.854 | 0.807 | 0.877 |
| Random Forest | 0.846 | 0.762 | 0.888 |

**Table S3** Performances of logistic regression models in five-fold cross-validations based on all combinations of six imaging features. The models were sorted by AUC. Models not using features related to enhanced CTs are shown in italic. Accu, Accuracy; Sen, Sensitivity; Spe, Specificity; FNR, False Negative Rate.

| **Variable combination** | | **AUC** | **Accu** | **Sen** | **Precision** | **Spe** | **FNR** |
| --- | --- | --- | --- | --- | --- | --- | --- |
| CTpre+Shape+N/C | | 0.919  ±0.003 | 0.859  ±0.005 | 0.734  ±0.012 | 0.822  ±0.008 | 0.921  ±0.002 | 0.266  ±0.012 |
| SD+CTpre+Shape+N/C | | 0.918  ±0.003 | 0.861  ±0.006 | 0.739  ±0.018 | 0.819  ±0.005 | 0.92  ±0 | 0.261  ±0.018 |
| CTpre+CTpost+Shape+N/C | | 0.918  ±0.003 | 0.854  ±0.006 | 0.722  ±0.008 | 0.823  ±0.011 | 0.92  ±0.005 | 0.278  ±0.008 |
| CTpre+N/C | | 0.918  ±0.002 | 0.858  ±0.005 | 0.726  ±0.01 | 0.828  ±0.01 | 0.924  ±0.003 | 0.274  ±0.01 |
| CTpre+CTpost+Shape+Homo+N/C | | 0.917  ±0.004 | 0.859  ±0.002 | 0.746  ±0.01 | 0.817  ±0.006 | 0.915  ±0.004 | 0.254  ±0.01 |
| CTpre+Homo+N/C | | 0.917  ±0.004 | 0.854  ±0.008 | 0.753  ±0.012 | 0.802  ±0.015 | 0.903  ±0.007 | 0.247  ±0.012 |
| CTpre+Shape+Homo+N/C | | 0.917  ±0.002 | 0.858  ±0.005 | 0.749  ±0.024 | 0.81  ±0.01 | 0.912  ±0.007 | 0.251  ±0.024 |
| CTpre+CTpost+N/C | | 0.915  ±0.005 | 0.854  ±0.006 | 0.718  ±0.013 | 0.817  ±0.012 | 0.92  ±0.007 | 0.282  ±0.013 |
| SD+CTpre+Shape+Homo+N/C | | 0.915  ±0.004 | 0.858  ±0.012 | 0.745  ±0.026 | 0.815  ±0.014 | 0.913  ±0.006 | 0.255  ±0.026 |
| SD+CTpre+N/C | | 0.915  ±0.004 | 0.858  ±0.003 | 0.731  ±0.009 | 0.824  ±0.006 | 0.92  ±0 | 0.269  ±0.009 |
| CTpre+CTpost+Homo+N/C | | 0.914  ±0.005 | 0.853  ±0.006 | 0.746  ±0.01 | 0.803  ±0.015 | 0.905  ±0.008 | 0.254  ±0.01 |
| SD+CTpre+CTpost+N/C | | 0.913  ±0.005 | 0.85  ±0.009 | 0.719  ±0.022 | 0.813  ±0.018 | 0.915  ±0.005 | 0.281  ±0.022 |
| SD+CTpre+CTpost+Shape+N/C | | 0.913  ±0.003 | 0.857  ±0.005 | 0.73  ±0.012 | 0.819  ±0.004 | 0.919  ±0.002 | 0.27  ±0.012 |
| SD+CTpre+CTpost+Shape+Homo+N/C | | 0.912  ±0.004 | 0.854  ±0.009 | 0.741  ±0.016 | 0.81  ±0.006 | 0.91  ±0.005 | 0.259  ±0.016 |
| SD+CTpre+Homo+N/C | | 0.911  ±0.005 | 0.85  ±0.007 | 0.741  ±0.018 | 0.793  ±0.01 | 0.903  ±0.007 | 0.259  ±0.018 |
| SD+CTpre+CTpost+Homo+N/C | | 0.91  ±0.003 | 0.851  ±0.009 | 0.743  ±0.017 | 0.794  ±0.017 | 0.903  ±0.009 | 0.257  ±0.017 |
| SD+CTpost+Shape+N/C | | 0.89  ±0.002 | 0.85  ±0.004 | 0.711  ±0.005 | 0.817  ±0.004 | 0.918  ±0.003 | 0.289  ±0.005 |
| *SD+CTpre+Shape+Homo* | | *0.89*  *±0.002* | *0.824*  *±0.005* | *0.748*  *±0.016* | *0.728*  *±0.007* | *0.861*  *±0.003* | *0.252*  *±0.016* |
| *SD+CTpre+Shape* | | *0.889*  *±0.006* | *0.819*  *±0.008* | *0.714*  *±0.016* | *0.732*  *±0.016* | *0.871*  *±0.007* | *0.286*  *±0.016* |
| CTpre+CTpost+Shape+Homo | | 0.888  ±0.004 | 0.826  ±0.004 | 0.744  ±0.014 | 0.737  ±0.008 | 0.865  ±0.002 | 0.256  ±0.014 |
| *CTpre+Shape+Homo* | | *0.887*  *±0.002* | *0.821*  *±0.012* | *0.736*  *±0.033* | *0.731*  *±0.014* | *0.863*  *±0.006* | *0.264*  *±0.033* |
| SD+CTpost+Shape+Homo+N/C | | 0.886  ±0.004 | 0.849  ±0.003 | 0.715  ±0.005 | 0.809  ±0.006 | 0.915  ±0.004 | 0.285  ±0.005 |
| SD+CTpre+CTpost+Shape+Homo | | 0.886  ±0.004 | 0.819  ±0.004 | 0.736  ±0.01 | 0.721  ±0.009 | 0.86  ±0.003 | 0.264  ±0.01 |
| CTpost+Shape+Homo+N/C | | 0.886  ±0.002 | 0.851  ±0.002 | 0.718  ±0.009 | 0.813  ±0.005 | 0.917  ±0.003 | 0.282  ±0.009 |
| *CTpre+Homo* | | *0.883*  *±0.003* | *0.804*  *±0.003* | *0.677*  *±0.009* | *0.717*  *±0.009* | *0.867*  *±0.004* | *0.323*  *±0.009* |
| CTpost+Shape+N/C | | 0.882  ±0.005 | 0.848  ±0.004 | 0.706  ±0.011 | 0.808  ±0.005 | 0.917  ±0.003 | 0.294  ±0.011 |
| *SD+CTpre+Homo* | | *0.882*  *±0.004* | *0.811*  *±0.007* | *0.706*  *±0.016* | *0.732*  *±0.016* | *0.863*  *±0.004* | *0.294*  *±0.016* |
| SD+Shape+Homo+N/C | | 0.882  ±0.002 | 0.851  ±0.002 | 0.714  ±0.006 | 0.813  ±0.004 | 0.919  ±0.002 | 0.286  ±0.006 |
| SD+CTpre+CTpost+Homo | | 0.881  ±0.007 | 0.804  ±0.008 | 0.681  ±0.023 | 0.711  ±0.012 | 0.864  ±0.006 | 0.319  ±0.023 |
| CTpre+CTpost+Homo | | 0.881  ±0.005 | 0.799  ±0.004 | 0.668  ±0.017 | 0.707  ±0.008 | 0.864  ±0.003 | 0.332  ±0.017 |
| SD+Shape+N/C | | 0.88  ±0.005 | 0.853  ±0.002 | 0.716  ±0.002 | 0.819  ±0.007 | 0.92  ±0 | 0.284  ±0.002 |
| SD+CTpre+CTpost+Shape | | 0.88  ±0.003 | 0.818  ±0.01 | 0.705  ±0.031 | 0.737  ±0.015 | 0.873  ±0.006 | 0.295  ±0.031 |
| SD+CTpost+N/C | | 0.879  ±0.003 | 0.856  ±0.004 | 0.729  ±0.008 | 0.82  ±0.011 | 0.919  ±0.002 | 0.271  ±0.008 |
| *SD+CTpre* | | *0.878*  *±0.004* | *0.801*  *±0.007* | *0.652*  *±0.017* | *0.719*  *±0.017* | *0.873*  *±0.004* | *0.348*  *±0.017* |
| SD+N/C | | 0.878  ±0.003 | 0.854  ±0 | 0.718  ±0.001 | 0.82  ±0.003 | 0.921  ±0 | 0.282  ±0.001 |
| SD+CTpost+Homo+N/C | | 0.877  ±0.003 | 0.857  ±0 | 0.739  ±0.002 | 0.815  ±0.01 | 0.915  ±0 | 0.261  ±0.002 |
| CTpost+Homo+N/C | | 0.876  ±0.003 | 0.852  ±0.002 | 0.726  ±0.006 | 0.807  ±0.005 | 0.915  ±0 | 0.274  ±0.006 |
| CTpost+N/C | | 0.872±0.004 | 0.851±0.005 | 0.71±0.016 | 0.823±0.008 | 0.92±0.001 | 0.29±0.016 |
| SD+CTpre+CTpost | | 0.871±0.004 | 0.795±0.008 | 0.651±0.023 | 0.714±0.017 | 0.866±0.004 | 0.349±0.023 |
| SD+Homo+N/C | | 0.867±0.011 | 0.852±0.002 | 0.715±0.006 | 0.817±0.002 | 0.92±0 | 0.285±0.006 |
| Shape+Homo+N/C | | 0.864  ±0.005 | 0.851  ±0.002 | 0.711  ±0.006 | 0.819  ±0.003 | 0.921  ±0 | 0.289  ±0.006 |
| SD+CTpost+Shape+Homo | | 0.863  ±0.004 | 0.801  ±0.006 | 0.653  ±0.024 | 0.726  ±0.009 | 0.873  ±0.005 | 0.347  ±0.024 |
| SD+CTpost+Shape | | 0.86  ±0.001 | 0.789  ±0.007 | 0.58  ±0.019 | 0.735  ±0.013 | 0.893  ±0.002 | 0.42  ±0.019 |
| *SD+Shape+Homo* | | *0.858*  *±0.002* | *0.781*  *±0.006* | *0.654*  *±0.02* | *0.682*  *±0.017* | *0.844*  *±0.011* | *0.346*  *±0.02* |
| SD+CTpost+Homo | | 0.853  ±0.007 | 0.808  ±0.009 | 0.674  ±0.008 | 0.732  ±0.018 | 0.873  ±0.012 | 0.326  ±0.008 |
| *CTpre+Shape* | | *0.85*  *±0.009* | *0.786*  *±0.005* | *0.614*  *±0.017* | *0.713*  *±0.009* | *0.872*  *±0.007* | *0.386*  *±0.017* |
| Homo+N/C | | 0.847  ±0.002 | 0.854  ±0 | 0.718  ±0.001 | 0.819  ±0.004 | 0.92  ±0.001 | 0.282  ±0.001 |
| *SD+Homo* | | *0.846*  *±0.005* | *0.796*  *±0.007* | *0.688*  *±0.013* | *0.705*  *±0.016* | *0.849*  *±0.008* | *0.312*  *±0.013* |
| *SD+Shape* | | *0.846*  *±0.003* | *0.776*  *±0.004* | *0.533*  *±0.012* | *0.722*  *±0.011* | *0.896*  *±0.003* | *0.467*  *±0.012* |
| Shape+N/C | | 0.846  ±0.002 | 0.854  ±0 | 0.716  ±0.002 | 0.816  ±0.002 | 0.92  ±0 | 0.284  ±0.002 |
| CTpre+CTpost+Shape | | 0.844  ±0.006 | 0.781  ±0.007 | 0.608  ±0.017 | 0.689  ±0.011 | 0.865  ±0.006 | 0.392  ±0.017 |
| CTpost+Shape+Homo | | 0.844  ±0.003 | 0.784  ±0.005 | 0.659  ±0.006 | 0.689  ±0.006 | 0.845  ±0.01 | 0.341  ±0.006 |
| SD+CTpost | | 0.844  ±0.001 | 0.781  ±0.009 | 0.561  ±0.005 | 0.728  ±0.019 | 0.889  ±0.011 | 0.439  ±0.005 |
| CTpost+Homo | | 0.835  ±0.004 | 0.76  ±0.003 | 0.656  ±0.014 | 0.635  ±0.01 | 0.811  ±0.006 | 0.344  ±0.014 |
| *SD* | | *0.835*  *±0.002* | *0.757*  *±0.01* | *0.495*  *±0.032* | *0.69*  *±0.025* | *0.886*  *±0.003* | *0.505*  *±0.032* |
| *CTpre* | | *0.831*  *±0.004* | *0.751*  *±0.008* | *0.5*  *6±0.023* | *0.645*  *±0.031* | *0.845*  *±0.004* | *0.44*  *±0.023* |
| CTpre+CTpost | | 0.823  ±0.005 | 0.746  ±0.013 | 0.554  ±0.035 | 0.635  ±0.024 | 0.841  ±0.007 | 0.446  ±0.035 |
| N/C | | 0.819  ±0.001 | 0.854  ±0 | 0.719  ±0.003 | 0.818  ±0.004 | 0.92  ±0 | 0.281  ±0.003 |
| *Shape+Homo* | | *0.814*  *±0.001* | *0.749*  *±0.017* | *0.76*  *±0.099* | *0.6*  *±0.021* | *0.743*  *±0.022* | *0.24*  *±0.099* |
| *Homo* | | *0.778*  *±0.001* | *0.761*  *±0* | *0.827*  *±0.003* | *0.6*  *±0.003* | *0.728*  *±0* | *0.173*  *±0.003* |
| CTpost+Shape | | 0.708  ±0.007 | 0.722  ±0.006 | 0.318  ±0.018 | 0.682  ±0.038 | 0.921  ±0.01 | 0.682  ±0.018 |
| CTpost | | 0.668  ±0.003 | 0.701  ±0.008 | 0.224  ±0.019 | 0.626  ±0.034 | 0.934  ±0.005 | 0.776  ±0.019 |
| *Shape* | *0.611*  *±0.001* | | *0.654*  *±0.004* | *0.216*  *±0.073* | *NA* | *0.869*  *±0.033* | *0.784*  *±0.073* |

**Table S4**: Comparison between models using AUC test. For selection of the most concise model possible, only the top model that included four features (“SD+CTpre+Shape+N/C”) and those top 6 models with three or fewer features were selected for comparison. As shown in the table below, the model with features “Ctpre + Shape + Homo”, was observed to be inferior to the other models with statistical significance. The best model, with features “CTpre + Shape + N/C”, is shown in bold.

| Model A | Model B | P-value in AUC test |
| --- | --- | --- |
| SD+CTpre+Shape+N/C | **CTpre+Shape+N/C** | 0.969 |
| SD+CTpre+Shape+N/C | SD+CTpre+N/C | 0.493 |
| SD+CTpre+Shape+N/C | CTpre+Homo+N/C | 0.351 |
| SD+CTpre+Shape+N/C | CTpre+N/C | 0.471 |
| SD+CTpre+Shape+N/C | CTpre+CTpost+N/C | 0.056 |
| SD+CTpre+Shape+N/C | CTpre+Shape+Homo | **0.012** |
| **CTpre+Shape+N/C** | SD+CTpre+N/C | 0.496 |
| **CTpre+Shape+N/C** | CTpre+Homo+N/C | 0.360 |
| **CTpre+Shape+N/C** | CTpre+N/C | 0.473 |
| **CTpre+Shape+N/C** | CTpre+CTpost+N/C | 0.059 |
| **CTpre+Shape+N/C** | CTpre+Shape+Homo | **0.012** |
| SD+CTpre+N/C | CTpre+Homo+N/C | 0.650 |
| SD+CTpre+N/C | CTpre+N/C | 0.961 |
| SD+CTpre+N/C | CTpre+CTpost+N/C | **0.009** |
| SD+CTpre+N/C | CTpre+Shape+Homo | **0.047** |
| CTpre+Homo+N/C | CTpre+N/C | 0.604 |
| CTpre+Homo+N/C | CTpre+CTpost+N/C | 0.208 |
| CTpre+Homo+N/C | CTpre+Shape+Homo | **0.028** |
| CTpre+N/C | CTpre+CTpost+N/C | **0.008** |
| CTpre+N/C | CTpre+Shape+Homo | **0.043** |
| CTpre+CTpost+N/C | CTpre+Shape+Homo | 0.146 |

**Table S5**: Comparison between models not using features related to enhanced CTs, using AUC test. For selection of the most concise model possible, only the top model that included four features (“SD+CTpre+Shape+Homo”) and those top 6 models with three or less features were selected for comparison. As is shown in the table below, the model, with features “SD + Shape + Homo”, was observed to be inferior to most other models with statistical significance. The best model, with features “CTpre + Shape + Homo”, is shown in bold.

| Model A | Model B | P-value in AUC test |
| --- | --- | --- |
| SD+CTpre+Shape+Homo | **CTpre+Shape+Homo** | 0.542 |
| SD+CTpre+Shape+Homo | SD+CTpre+Shape | 0.706 |
| SD+CTpre+Shape+Homo | CTpre+Homo | 0.226 |
| SD+CTpre+Shape+Homo | SD+CTpre+Homo | 0.205 |
| SD+CTpre+Shape+Homo | SD+CTpre | 0.195 |
| SD+CTpre+Shape+Homo | SD+Shape+Homo | **0.006** |
| **CTpre+Shape+Homo** | SD+CTpre+Shape | 0.973 |
| **CTpre+Shape+Homo** | CTpre+Homo | 0.349 |
| **CTpre+Shape+Homo** | SD+CTpre+Homo | 0.456 |
| **CTpre+Shape+Homo** | SD+CTpre | 0.379 |
| **CTpre+Shape+Homo** | SD+Shape+Homo | **0.020** |
| SD+CTpre+Shape | CTpre+Homo | 0.610 |
| SD+CTpre+Shape | SD+CTpre+Homo | 0.575 |
| SD+CTpre+Shape | SD+CTpre | 0.157 |
| SD+CTpre+Shape | SD+Shape+Homo | **0.025** |
| CTpre+Homo | SD+CTpre+Homo | 0.954 |
| CTpre+Homo | SD+CTpre | 0.658 |
| CTpre+Homo | SD+Shape+Homo | 0.076 |
| SD+CTpre+Homo | SD+CTpre | 0.564 |
| SD+CTpre+Homo | SD+Shape+Homo | 0.051 |
| SD+CTpre | SD+Shape+Homo | 0.160 |

**Table S6** Performances of M1 and M2 in five times of five-fold cross-validations. Accu, Accuracy; Sen, Sensitivity; Spe, Specificity; FNR, False Negative Rate. ^a^Standard deviation.

| **Variable combination** | **AUC** | **Accu** | **Sen** | **Precision** | **Spe** | **FNR** |
| --- | --- | --- | --- | --- | --- | --- |
| CTpre+Shape+N/C | 0.919  ±0.003^a^ | 0.859  ±0.005 | 0.734  ±0.012 | 0.822  ±0.008 | 0.921  ±0.002 | 0.266  ±0.012 |
| *CTpre+Shape+Homo* | *0.887*  *±0.002* | *0.821*  *±0.012* | *0.736*  *±0.033* | *0.731*  *±0.014* | *0.863*  *±0.006* | *0.264*  *±0.033* |

**Figure S1.** Pearson Correlation Coefficients between quantitative features, including age, long diameter (LD), short diameter (SD), CT value on pre-enhanced images (CTpre), and CT value on post-enhanced images (CTpost).


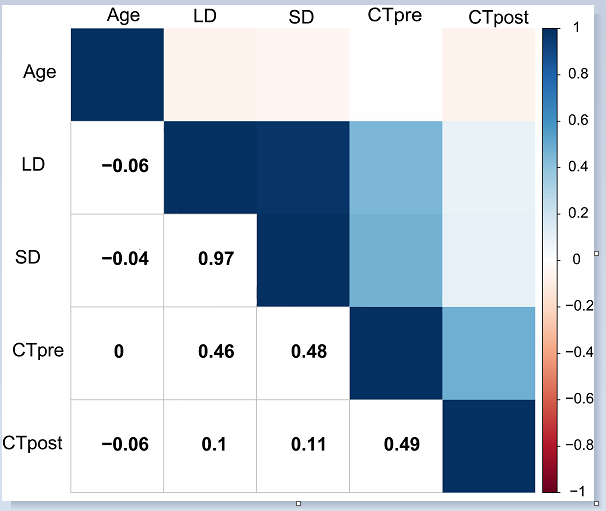


**Figure S2**. Spearman Correlation Coefficients between qualitative features, including gender, shape, Homogeneity (Homo), Necrosis or Cystic degeneration (N/C), Calcification (Calc), and Contour.


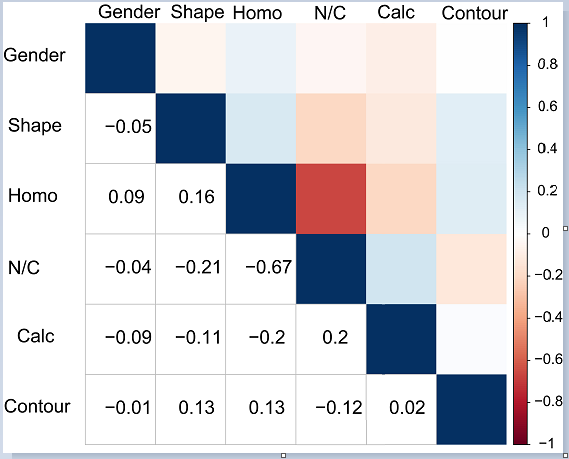


**Figure S3**. ROC curves for the original S1 scoring system (solid) and the simplified scoring system (dashed). There was no significant difference between the AUCs based on the AUC test.


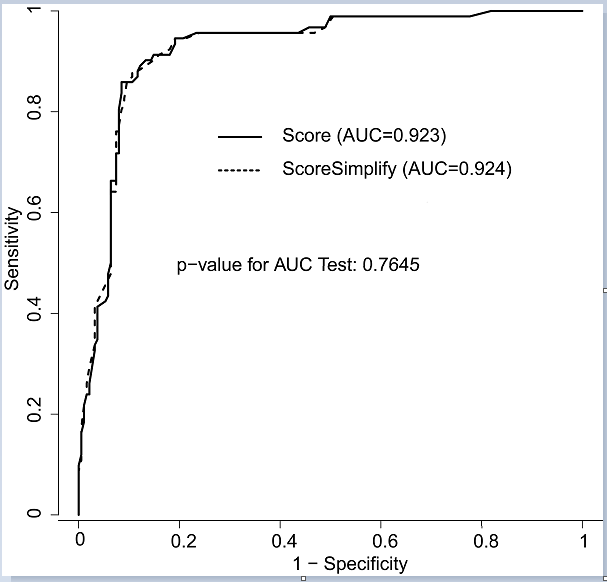


**Figure S4**. The dynamics changes of AUCs as the variation of cutoff values in S1 in the leave-one-out test


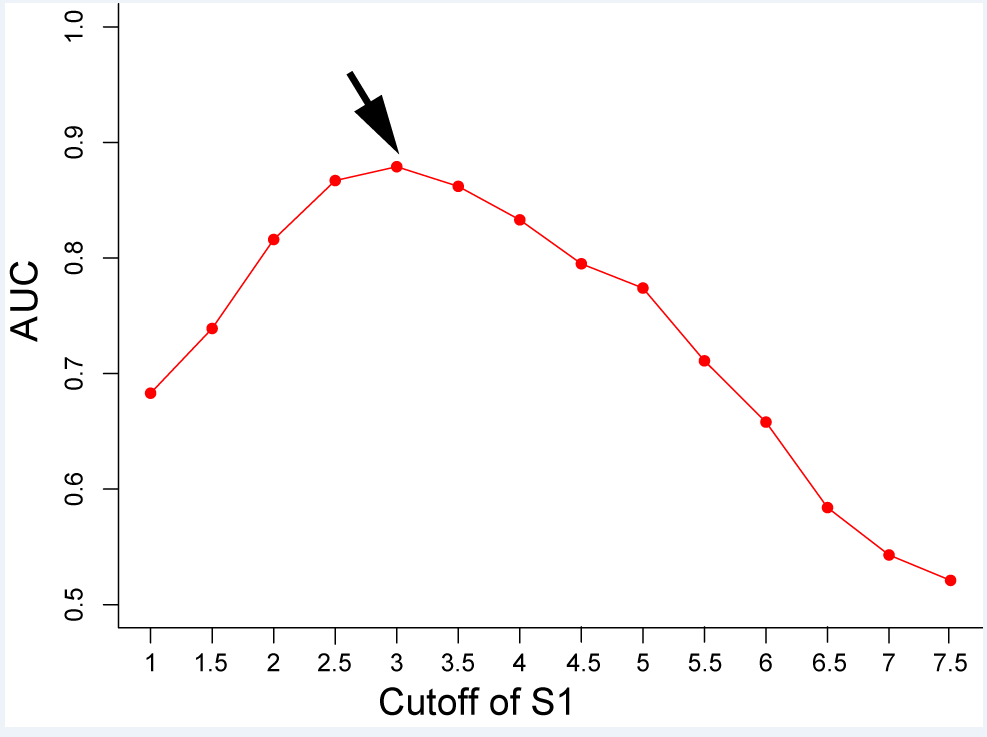


**Figure S5.** ROC curves for the original S2 scoring system (solid) and the simplified S2 scoring system (dashed). There was no significant difference between the AUCs based on the AUC test.


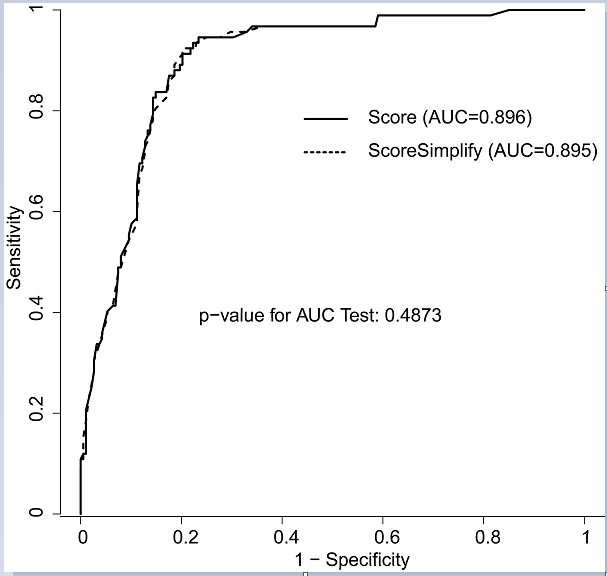


**Figure S6.** The dynamics changes of AUCs as the variation of cutoff values in S2 score in the leave-one-out test


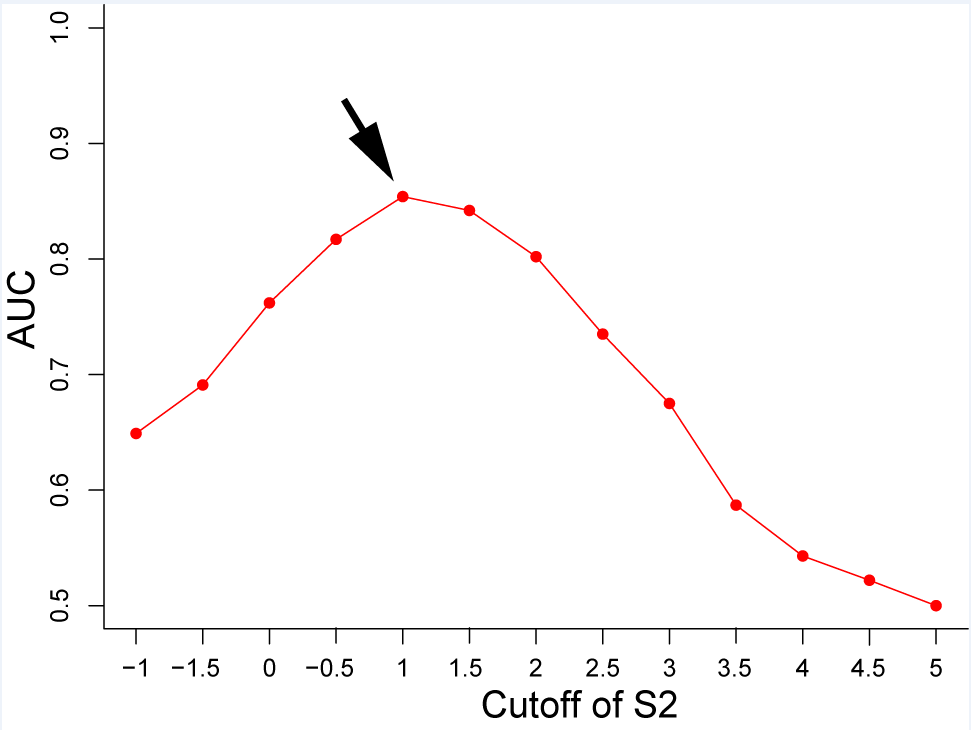

Supplement: Supplementary file 1 [file DataSheet_1.docx]
